# HIV-1 Vpr activates the G_2 _checkpoint through manipulation of the ubiquitin proteasome system

**DOI:** 10.1186/1743-422X-4-57

**Published:** 2007-06-08

**Authors:** Jason L DeHart, Erik S Zimmerman, Orly Ardon, Carlos MR Monteiro-Filho, Enrique R Argañaraz, Vicente Planelles

**Affiliations:** 1 Division of Cell Biology and Immunology, Department of Pathology, University of Utah School of Medicine, 15 North Medical Drive East #2100 – Room 2520, Salt Lake City, UT 84112, USA; 2 Laboratório de Farmacologia Molecular (CP 04536), Faculdade de Saude, Universidade de Brasília, 70919-970 Brasília, DF, Brazil

## Abstract

HIV-1 Vpr is a viral accessory protein that activates ATR through the induction of DNA replication stress. ATR activation results in cell cycle arrest in G_2 _and induction of apoptosis. In the present study, we investigate the role of the ubiquitin/proteasome system (UPS) in the above activity of Vpr. We report that the general function of the UPS is required for Vpr to induce G_2 _checkpoint activation, as incubation of Vpr-expressing cells with proteasome inhibitors abolishes this effect. We further investigated in detail the specific E3 ubiquitin ligase subunits that Vpr manipulates. We found that Vpr binds to the DCAF1 subunit of a cullin 4a/DDB1 E3 ubiquitin ligase. The carboxy-terminal domain Vpr(R80A) mutant, which is able to bind DCAF1, is inactive in checkpoint activation and has dominant-negative character. In contrast, the mutation Q65R, in the leucine-rich domain of Vpr that mediates DCAF1 binding, results in an inactive Vpr devoid of dominant negative behavior. Thus, the interaction of Vpr with DCAF1 is required, but not sufficient, for Vpr to cause G_2 _arrest. We propose that Vpr recruits, through its carboxy terminal domain, an unknown cellular factor that is required for G_2_-to-M transition. Recruitment of this factor leads to its ubiquitination and degradation, resulting in failure to enter mitosis.

## Background

The HIV-1 encoded viral protein R induces cell cycle arrest and apoptosis through activation of the serine/threonine kinase known as the ataxia telangiectasia-mutated and Rad3-related (ATR) protein [1,2]. Vpr activates ATR by inducing replication stress, a cellular condition that occurs in dividing cells as a consequence of deoxyribonucleotide depletion, stalled replication forks, or ultraviolet light-induced DNA damage. How Vpr induces replication stress remains uncertain.

Cell cycle progression is tightly regulated by several mechanisms, including orchestrated destruction of cell cycle mediators, their phosphorylation/de-phosphorylation and their subcellular localization. Destruction of cell cycle regulators is typically mediated by the proteasome and involves polyubiquitination by E3 ubiquitin ligases. The existence of a connection between proteasomal degradation of cell cycle regulators and ATR activation is exemplified in several instances involving Cdt1 [3-5] and Chk1 [6] among others.

Certain viral proteins are known to bind to the substrate specificity subunits of E3 ligases to redirect specificity to non-cognate targets. Examples of these viral proteins include hepatitis B protein X [7], human papilloma virus E6 [8], simian virus 5 V protein [9,10], HIV-1 Vif [11-13], and HIV-1 Vpu [14]. In the present study, we examined in detail the potential role of the UPS in the ability of HIV-1 Vpr to induce G_2 _arrest.

## Results and Discussion

### Proteasome inhibitors relieve Vpr-induced G_2 _arrest

Several lines of evidence suggest a possible functional interaction of Vpr with the UPS. First, a protein known as RIP, that was discovered as an interaction partner of Vpr [15], was recently shown to be part of a family of WD-repeat proteins that are found in association with cullin 4a/DDB1 E3 ubiquitin ligases [9]. Accordingly, RIP was recently renamed DDB1-Cul4A-associated factor-1, DCAF1 [9]. Second, Vpr was recently found to induce degradation of uracil-N-glycoslylase (UNG) through the UPS [16]. Finally, post transcriptional silencing of the damaged DNA-binding protein 1 (DDB1) leads to cell cycle arrest at the G_2_-to-M transition [3]. Therefore, we set out to directly evaluate the role of the UPS in Vpr induced G_2 _arrest. We resorted to two different methods of proteasome inhibition: incubation with epoxomicin, and over-expression of a dominant-negative ubiquitin mutant, Ub(K48R) [17] that blocks formation of polyubiquitin chain conjugates. Cells were either incubated with epoxomicin, DMSO, or transfected with Ub(K48R) or empty vector. To induce Vpr expression, we transduced HeLa cells with the Vpr-expressing lentivirus vector, pHR-VPR-IRES-GFP [2,18], and analyzed the cell cycle profile 48 post transduction. The vector pHR-VPR-IRES-GFP expresses Vpr in the absence of all other HIV-1 genes, and also expresses GFP via an internal ribosome entry site [19]. For simplicity, we will refer to this lentiviral vector as pHR-VPR. Throughout this work, we measured GFP expression by flow cytometry and HA-Vpr expression by WB, to verify that levels of infection with lentiviral vectors were not affected by the various treatments (inhibitors, siRNAs and dominant-negative constructs).

**Figure 1:**
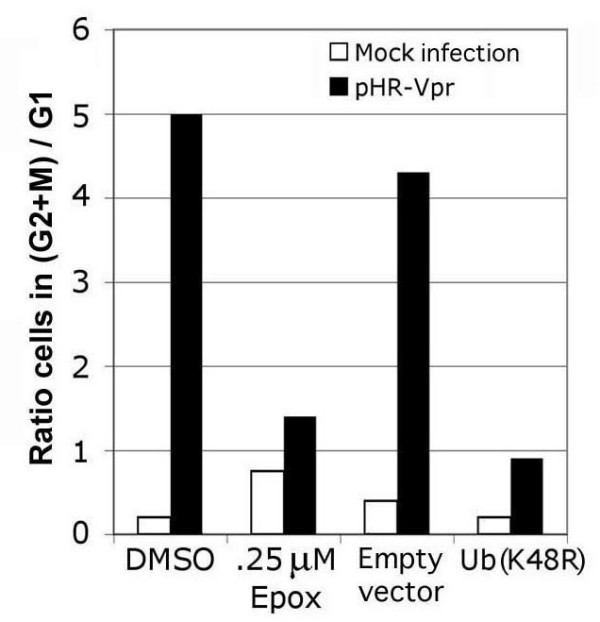
Role of the ubiquitin proteasome system in Vpr-induced G_2 _arrest. Incubation with epoxomicin or overexpression of Ub(K48R) block Vpr induced G_2 _arrest when induced by Vpr, but not when induced by the topoisomerase inhibitor, etoposide.

Incubation with epoxomicin induced a small, basal level of G_2 _arrest in non-Vpr expressing cells. Strikingly, however, epoxomicin incubation dramatically relieved Vpr-induced G_2 _arrest (Figure 1; cell cycle profile data are presented in Additional file 1). In agreement with the epoxomicin results, over-expression of Ub(K48R) also very effectively abolished the induction of G_2 _arrest in Vpr-expressing cells (Figure 1). Therefore, we conclude that Vpr function requires the activity of the UPS. On the other hand, because the above proteasome inhibitors do not provide any information on the specific ubiquitin ligases involved, we next examined the potential E3 ligase components that are relevant to Vpr.

### Affinity chromatography and mass spectrometry identify DCAF1 as a potential interactor of Vpr

**Figure 2:**
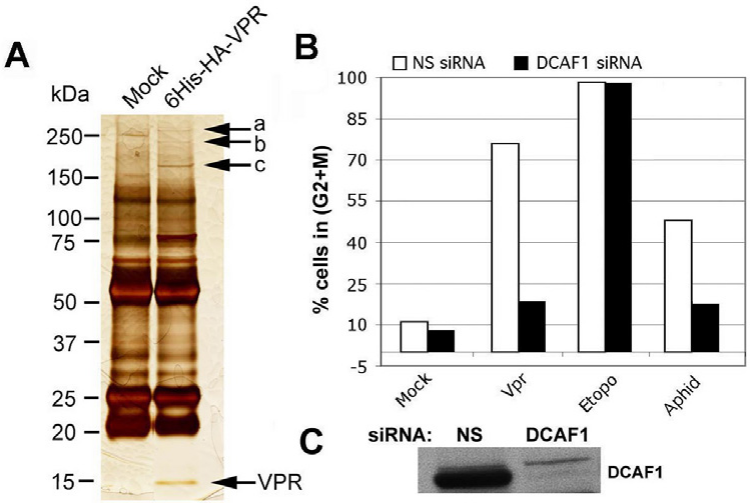
Role of DCAF1 in Vpr-induced G_2 _arrest. A. Identification of Vpr-interacting proteins by affinity chromatography followed by mass spectrometry; band labeled as "c" was identified as being DCAF1. B. Knockdown of DCAF1 abolishes Vpr function. C. Western blot demonstrates efficient knockdown of DCAF1. NS, non-specific siRNA.

In an effort to identify cellular proteins that may interact with Vpr to mediate its function, we performed affinity chromatography followed by mass spectrometry. 293FT cells were transfected with a vector encoding a hexa-histidine and hemagglutinin-tagged Vpr construct (pHR-His-HA-VPR-IRES-GFP), or mock-transfected, and then lysed at 24 hours. Lysates were bound to a Ni-NTA agarose column. Bound proteins were eluted and then immunoprecipitated with an anti-HA antibody followed by protein G agarose beads, boiled and resolved on SDS-PAGE. The resulting gel was silver-stained (Figure 2, panel A). We observed three high-molecular weight bands (labeled "a, b and c") present in the Vpr lane but not in the control lane (Figure 2, panel A). Bands a, b, and c were excised, trypsin digested, and analyzed by mass spectrometry. Band c was identified as DCAF1 [9], and was recently reported by Le Rouzic et al. to interact with Vpr [20]. Bands a and b could not be identified.

### DCAF1 is required for induction of G2 arrest by HIV-1 Vpr

To test whether DCAF1 plays a role in Vpr-induced G_2 _arrest, we performed knockdown of DCAF1, and then transduced cells with pHR-VPR. Knockdown of DCAF1 did not affect the cell cycle profile of mock-transduced cells, but almost completely relieved the induction of G_2 _arrest by Vpr (Figure 2, panel B; cell cycle profile panels are presented in Additional file 2). To test whether the requirement for DCAF1 toward induction of G_2 _arrest is general or specific for Vpr we performed a parallel experiment with etoposide, a topoisomerase II inhibitor that induces double-strand breaks. Knockdown of DCAF1 had no effect on etoposide-induced G_2 _checkpoint activation (Figure 2, panel B). Knockdown of DCAF1 was verified by WB using a rabbit polyclonal antiserum kindly provided by Dr. Ling-Jun Zhao, Saint Louis University [15] (Figure 2, panel C).

Etoposide generates double-strand breaks and activates the G_2 _checkpoint through a combination of pathways that activate ATM, ATR and/or DNA-PK. However, Vpr specifically activates ATR only [1,21]. Low-dose aphidicolin induces mild DNA polymerase inhibition and results in specific activation of ATR [22]. Therefore, we tested whether checkpoint activation by low dose aphidicolin could also be abrogated by DCAF1 knockdown. As shown in Figure 2, panel B, DCAF1 knockdown effectively relieved chekckpoint activation.

We conclude that DCAF1 is specifically required for checkpoint activation by Vpr and aphidicolin, but not by the DNA damaging agent, etoposide. Since Vpr and aphidicolin both activate ATR, our results suggest, although do not demonstrate, the possibility that the presence of DCAF1 may be normally required for ATR activation.

### DCAF1 brings HIV-1 Vpr in association with Cullin4/DDB1

Since the presence of DCAF1 is required for Vpr function, we decided to test whether Vpr interacts with DCAF1. In addition, because DCAF1 is known to function in the context of DDB1 [9,23,24], which bridges DCAF1 to cullin 4, we also asked whether Vpr can be found in association with DDB1.

**Figure 3:**
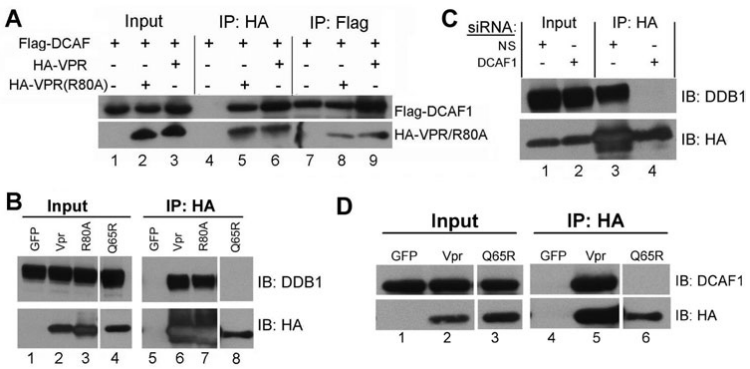
Co-immunoprecipitation studies with Vpr, DCAF1 and DDB1. A. Flag-DCAF1 was cotransfected into HeLa cells with either HA-Vpr or HA-Vpr(R80A). Immunoprecipitation was performed with either HA or Flag antibody as indicated. Immunoprecipitates were analyzed by WB for the presence of Flag-DCAF1 or HA-Vpr. B. GFP, HA-Vpr, HA-Vpr(R80A), or HA-Vpr(Q65R) constructs were transfected, and immunoprecipitations with HA antibody were performed. Immunoprecipitates were analyzed by WB with an antibody against endogenous DDB1. C. Knockdown of DCAF1 abolishes the association between Vpr and DDB1; HeLa cells were transected with HA-Vpr, in the presence of non-specific (NS) or DCAF1-specific siRNA. 48 hours later, cell extracts were immunoprecipitated with HA antibody and analyzed by WB for the presence of endogenous DDB1. D. HA-Vpr(Q65R) fails to interact with DCAF1.

We transfected a Flag-DCAF1 construct along with either HA-Vpr or HA-Vpr(R80A), a Vpr mutant that is incapable of inducing G_2 _arrest [2,18,25] and, 48 hours later, we immunoprecipitated Flag-DCAF1 from cell extracts. When immunoprecipitates obtained with anti-HA antibody (specific for HA-Vpr) were analyzed by WB for the presence of Flag-DCAF1 (Figure 3, panel A), the presence of a reactive band of the expected molecular weight was evident both for HA-Vpr(R80A) (lane 5) and HA-Vpr (lane 6). This immunoprecipitation was reproduced when performed in the reciprocal order (lanes 8 and 9). From these experiments, we conclude that Vpr and DCAF1 physically interact.

In separate experiments, we also detected an interaction between Vpr – and also Vpr(R80A) – and myc-tagged cullin 4a (data not shown). These observations confirm that Vpr targets a cullin 4-based E3 ligase, for which DCAF1 is a cognate substrate specificity receptor [9,23,24].

DCAF1 is linked to cullin 4a via DDB1 [9]. Thus, we wished to test whether Vpr could also be found in association with DDB1. We demonstrated co-immunoprecipitation of Vpr and, separately, Vpr(R80A), with DDB1 (Figure 3, panel B, lanes 6 and 7). Since the inactive mutant, Vpr(R80A), binds to DDB1 and DCAF1 with similar efficiency as wild-type Vpr, we conclude that binding to DCAF1/DDB1 is not sufficient for Vpr function.

**Figure 4:**
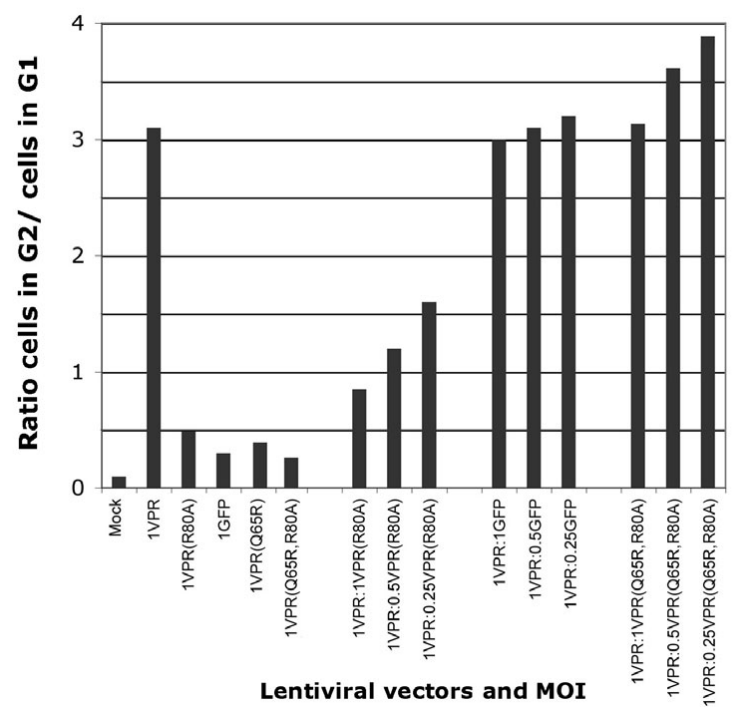
Vpr(R80A) functions as a dominant-negative molecule. HeLa cells were infected with a constant MOI (MOI = 1) of pHR-VPR vector, and a variable MOI (1, 0.5, 0.25) of pHR-GFP, pHR- Vpr(R80A) or pHR-VPR(Q65R, R80A) as indicated. Cell cycle profiles were evaluated at 48 hours post transduction.

In order to generate a more appropriate negative control for IP experiments, we constructed the mutation Vpr(Q65R), which disrupts a leucine-rich region required for binding to DCAF1 [15]. Vpr(Q65R) failed to associate with DDB1 (Figure 3, panel B, lane 8), DCAF1 (Figure 3, panel D, lane 6), and also failed to induce G_2 _arrest (Figure 4).

From the above results, we conclude that binding to DCAF1/DDB1 is required for Vpr function, but it is not sufficient. The above experiments, however, could not discern whether Vpr actually binds to DCAF1, DDB1, or both. To further characterize these interactions, we performed knockdown of DCAF1, and asked whether Vpr could be co-precipitated with DDB1 in the absence of DCAF1. While transfection of a non-specific siRNA did not affect pull down of DDB1 with Vpr (Figure 3 panel C, lane 3), transfection of DCAF1-specific siRNA abolished any detectable pull down of DDB1 (lane 4). Based on these results, we propose that Vpr, DCAF1 and DDB1 form a ternary complex in which is DCAF1 acts to bridge Vpr and DDB1.

### Vpr(R80A) acts as a dominant-negative protein

Based on the model that Vpr binds to DCAF1/DDB1 to trigger ubiquitination of a certain cellular target, one could envision two types of inactive Vpr mutants. The first category would include Vpr mutants that fail to bind to DCAF1. The second type of mutants would include those that retain the ability to bind to DCAF1 but are unable to recruit the putative cellular target. We also predict that mutants of the second, but not the first type, would act as dominant-negative proteins.

The domain of Vpr that binds to DCAF1 was mapped by Zhao et al. [15] to the leucine-rich (LR) motif ^60^LIRILQQLL^68 ^of HIV-1_89.6 _Vpr. Vpr(R80A), while unable to induce G_2 _arrest [2,18,25], has an intact LR domain, which explains its ability to bind DCAF1 (Figure 3). The inability of Vpr(R80A) to induce G_2 _arrest could, therefore, be due to lack of recruitment of a potential target for ubiquitination. If this were true, then Vpr(R80A) should act as a dominant-negative mutant, and interfere with the function of wild-type Vpr by competing for binding to DCAF1.

To test the previous idea, we co-infected cells with a constant amount of pHR-Vpr vector (MOI = 1.0) and decreasing amounts of Vpr(R80A) (MOIs of 1, 0.5 and 0.25), and then assessed the cell cyle profile in these cultures (Figure 4). As a negative control, we performed a parallel experiment in which pHR-Vpr(R80A) was replaced by a vector expressing GFP only (see Additional file 3 for cell cycle profile data). Co-transduction of even small amounts of Vpr(R80A) vector resulted in strong reduction of Vpr induced G_2 _arrest, whereas transduction with equivalent infectious units of pHR-GFP had no effect.

We then hypothesized that if the dominant-negative activity of Vpr(R80A) stems from its ability to bind to DCAF1, then introducing the Q65R mutation in Vpr(R80A) would abolish the dominant-negative activity. Thus, we constructed the double mutant, Vpr(Q65R, R80A). Vpr(Q65R, R80A) was, as expected, unable to bind DCAF (data not shown), or to induce G_2 _arrest (Figure 4). Vpr(Q65R, R80A) did not behave as a dominant-negative protein (Figure 4; see also Additional file 3).

### Role of DDB1 in Vpr function

DDB1 is known to exert two different functions that require its participation in distinct molecular complexes. The DNA damage recognition of DDB1 involves binding to certain types of DNA damage, and then recruitment of the NER machinery [26]. This function of DDB1 requires the interaction with its partner molecule, DDB2/XPE, a WD-repeat protein that contains the intrinsic damaged DNA-binding ability of the DDB1/DDB2 complex [26]. On the other hand, DDB1 interacts with a number of WD repeat proteins (which include DCAF1 and DDB2/XPE among others) to form the substrate specificity module for cullin 4-type E3 ubiquitin ligases [9,23,24]. The natural target(s) for DDB1/DCAF1 are not known.

**Figure 5:**
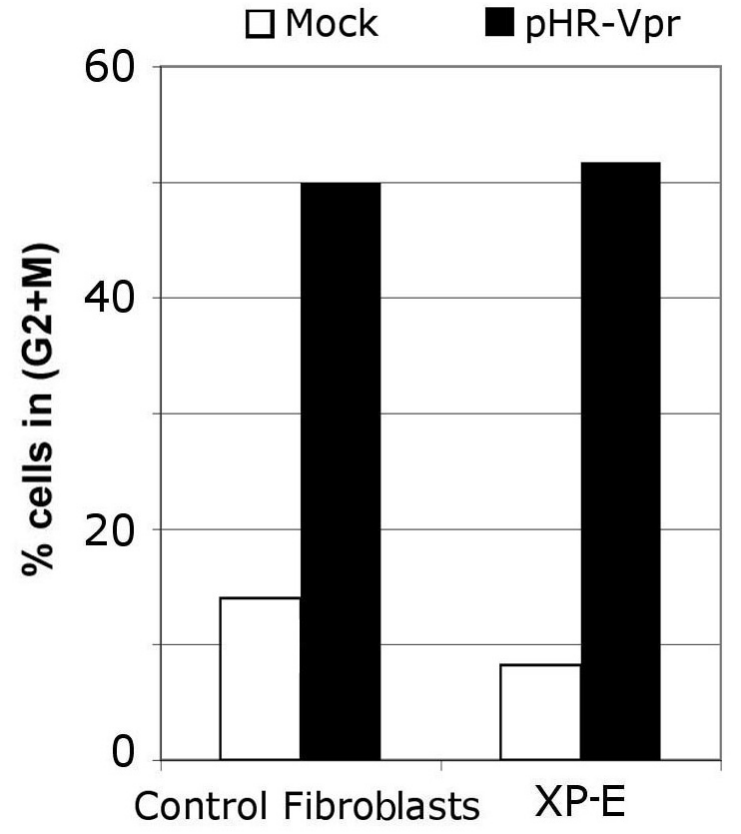
DDB2 is dispensable for Vpr function. Xeroderma pigmentosum complementation group E fibroblasts or control fibroblasts were infected with pHR-VPR or mock-infected and, 48 hours later, cell cycle profile was analyzed.

Our observation that proteasome inhibitors can block Vpr-induced G_2 _arrest suggests a role for proteasome-mediated degradation of a putative cellular factor required for the G_2_-to-M transition. This model suggests that Vpr subverts the second function of DDB1 (an E3 ubiquitin ligase specificity module) and not the first one (recognition of damaged DNA). To formally test the first function of DDB1 in the context of Vpr, we resorted to the use of cells from xeroderma pigmentosum complementation group E (XP-E), which lack DDB2/XPE function. As shown in Figure 5, XP-E cells arrest in G_2 _in response to Vpr expression, in a manner that is similar to that of control fibroblasts. These results, indicate that DDB2 is dispensable for the induction of G_2 _arrest by Vpr. Thus, these results, together with the finding that Vpr binds to DCAF1, support the notion that DDB1 works in concert with members of a Cullin 4 based E3 ubiquitin ligase.

### Vpr does not affect the steady-state levels of Cdt1

Cdt1 is an important component of the pre-replication complex as it mediates licensing of replication forks [27,28]. Upon DNA damage or firing of origins of replication, Cdt1 becomes ubiquitinated by the Cul4A-DDB1 E3 ligase complex resulting in its proteasomal degradation [28-30]. It was recently demonstrated that depletion of DDB1 from cells results in the stabilization of Cdt1 leading to re-replication and DNA damage. This results in activation of the G_2 _checkpoint [3].

**Figure 6:**
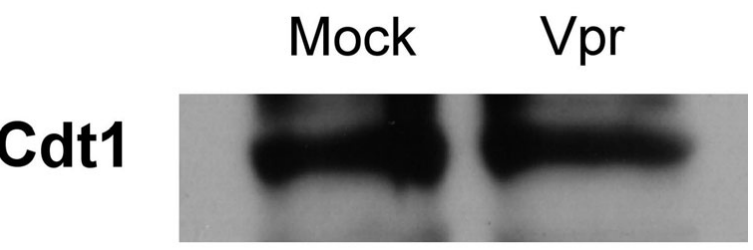
Changes in Cdt1 protein level are not associated with Vpr expression. Cells were transduced pHR-VPR or mock-transduced, and at 48 hours post-transduction, lysed and assayed for Cdt1 levels by WB.

Thus, is possible that Vpr interacts with the Cul4A-DDB1 E3 ligase complex in order to disrupt its normal function, leading to abnormal stabilization of Cdt1. If Vpr were acting in this manner, we would expect an increase in the steady-state levels of Cdt1 in the presence of Vpr. In order to test this idea, we transduced HeLa cells with pHR-VPR and monitored Cdt1 levels by WB. We found that Cdt1 levels did not change when compared to those of mock-transduced cells (Figure 6). Therefore, we conclude that Vpr does not activate the G_2 _checkpoint via inhibition of the Cul4A-DDB1 E3 ligase, which would result in failure do degrade Cdt1.

Vpr is the third HIV-1-enconded protein that has been reported to manipulate E3 ubiquitin ligases. Previous examples are Vpu, which induces degradation of CD4 [14], and Vif, which induces degradation of APOBEC3G and F [11-13]. Degradation of CD4 by Vpu frees nacent gp160 in the endoplasmic reticulum from interacting prematurely with the viral receptor. Destruction of APOBEC3G and F by Vif is necessary for the virus to avoid hypermutation via APOBEC deamination of cytidine residues. The cellular protein whose degradation leads to Vpr-induced G_2 _arrest is unknown. Therefore, it is difficult to speculate on the consequences that such degradation might play in the virus replication cycle.

Schrofelbauer et al. proposed a model in which Vpr binds directly to DDB1 and causes the DDB1/DDB2 complex to dissociate [31]. Dissociation of DDB1/DDB2 then leads to inability to recognize and repair DNA damage, and this DNA damage is the ultimate trigger of ATR activation and G_2 _arrest [31]. Our results are inconsistent with the previous model in that (a) the function of the DDB1/DDB2 complex in recognizing DNA damage does not require DCAF1, whereas Vpr induced G_2 _arrest does; (2) Vpr is unable to directly associate with DDB1; instead, Vpr binds to DCAF1; (3) the ability of the DDB1/DDB2 complex to bind to damaged DNA does not require a functional UPS, whereas Vpr function does; and (4) Vpr(R80A), although incapable of inducing G_2 _arrest, still interacts with DDB1.

**Figure 7:**
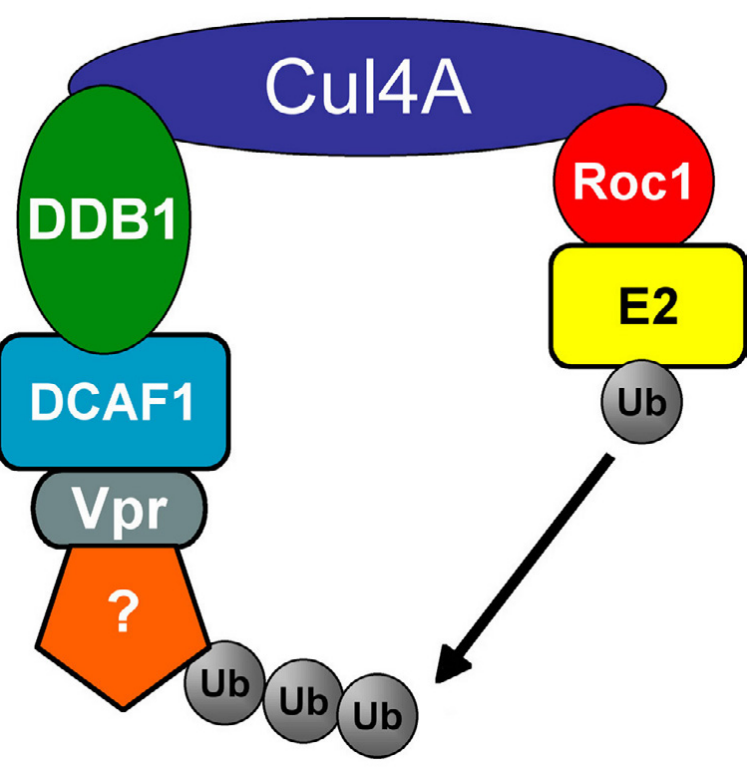
Proposed model for the interaction of Vpr with an E3 ubiquitin ligase. Question mark denotes putative degradation target.

On the other hand, our results confirm and extend the model recently proposed by Le Rouzic and collaborators [20]. This model, shown in Figure 7, proposes that interaction of Vpr with the E3 ubiquitin ligase complex is mediated by DCAF1. This model is essentially different from the one proposed by Angers et al. for the interaction of SV5 protein V with DDB1, in that protein V binds DDB1 directly and in a competitive manner with the DCAF subunit [9,32,33], whereas Vpr binds to DCAF1 and does not compete with its interaction with DDB1.

## Conclusion

In conclusion, our results strongly suggest a model in which Vpr manipulates a cullin 4/DDB1/DCAF1 E3 ubiquitin ligase complex, which in turn leads to degradation of an as yet unknown protein, and this leads to ATR activation. Future investigations will be directed at identifying this putative ubiquitination target, and how it functions to regulate cell cycle progression.

## Methods

### Affinity purification and identification of VPR-interacting proteins

293FT cells were transfected with vectors encoding His-HA-VPR (pHR-His-HA-VPR-IRES-GFP) or mock transfected by calcium phosphate transfection. Cells were harvested 24 hours after transfection and lysed in Ni-NTA binding buffer (0.5% NP-40, 20 mM Imidazole,100 mM NaCl, 20 mM NaH2PO4, pH 7.5) with protease inhibitor cocktail (Roche). Cell lysates were bound to 2 mL NiNTA agarose slurry (Qiagen) for 1 hour at 4°C. The Ni-NTA agarose column was washed with 4 column volumes of Ni-NTA binding buffer, and bound proteins were eluted in Ni-NTA binding buffer containing 150 mM Imidazole. Eluates were then immunoprecipitated with an anti-HA antibody (Covance) followed by protein G agarose beads (Santa Cruz). Immunoprecipitates were washed 3 times with Ni-NTA binding buffer, then boiled in SDS-PAGE loading buffer and resolved by SDS-PAGE. Gels were silver stained using the SilverQuest kit (Invitrogen) and protein bands of interest excised, trypsin digested, and analyzed by mass spectrometry.

### Cell Lines and Transfections

HEK293FT (Invitrogen, Carlsbad CA) and HeLa cells were maintained in Dulbelcco's Modified Eagle's Medium, supplemented with 10% FBS and 2 mM L-Glutamine. HEK293FT cells were transfected by either calcium phosphate [34] or Polyfect (Qiagen) according to manufacturer's instructions. HeLa cells were transfected with Oligofectamine as described previously [21].

### Plasmids

pCDNA3.1-Ubiquitin K48R was provided by M. Pagano. Flag-DCAF1 was purchased from GeneCopoeia, Inc., Germantown, MD. The Q65R mutation in Vpr was made in pHR-VPR using Quikchange II XL (Stratagene).

### siRNAs

Non-specific and DCAF1 siRNAs were purchased from Dharmacon. The following sequence was used to target DCAF1 CCACAGAAUUUGUUGCGCAUU [20].

### Drugs

Epoxomicin (Calbiochem) was solubilized in DMSO and used at 0.25 μM final concentration. Etoposide was purchased from Sigma and used at 10 μM final concentration.

### Immunoprecipitation and Western blot

IP and WB were performed as previously described [34]. DDB1 antibody was from ABCAM. Hemagglutinin-specific antibody for epitope tag detection, HA.11, was from Covance. FLAG (M2) was from Sigma. Dr. Ling-Jun Zhao (Saint Louis University) provided rabbit polyclonal serum against endogenous DCAF1.

### Cell cycle analyses

Cells were trypsinized, washed and fixed in cold 70% ethanol. Cells were then stained with propidium iodide and analyzed for DNA content as previously described [2]. ModFit was then used to analyze the cell cycle profiles.

### Lentiviral vectors

pHR-VPR-IRES-GFP (herein referred to as pHR-VPR), pHR-VPR(R80A)-IRES-GFP and pHR-GFP, were produced and titered as previously described [1,21]. Cells were infected by spin infection as follows. 10^6 ^cells were diluted in viral stocks with 10 μg/ml polybrene and centrifuged at 1,700 × g for 2 hours at 25°C, and cells were then washed and resuspended in normal growth medium.

## Abbreviations

Vpr: viral protein R; ATR: ataxia telangiectasia-mutated and Rad3-related protein; DCAF: DDB1-Cul4A-associated factor 1; DDB1 and DDB2: damaged DNA-binding proteins 1 and 2; WB: Western blot; MOI, multiplicity of infection); IP: immunoprecipitiation.

## Competing interests

The author(s) declare that they have no competing interests.

## Authors' contributions

JLD and ESZ performed most of the experimental work. OA, ERA and CMRM provided technical assistance. VP conceived and participated in the study design. All authors read and approved the manuscript.

## Supplementary Material

Additional file 1 Cell cycle profiles for experiments on the role of the ubiquitin proteasome system in Vpr-induced G_2 _arrest, corresponding to data shown in Figure 1. Click here for file

Additional file 2 Cell cycle profiles for experiments on the role of DCAF1 in Vpr-, etoposide- and aphidicolin-induced G_2 _arrest, corresponding to data shown in Figure 2B. Click here for file

Additional file 3 Cell cycle profiles for experiments showing the dominan-negative activity of Vpr(R80A), corresponding to data shown in Figure 4. Click here for file
