# Supplementary figures and images for: HIV-1 Vpr activates the G2 checkpoint through manipulation of the ubiquitin proteasome system
Source: Virol J. 2007 Jun 8;4:57. doi: 10.1186/1743-422X-4-57 (PMC1904188; doi:10.1186/1743-422X-4-57)

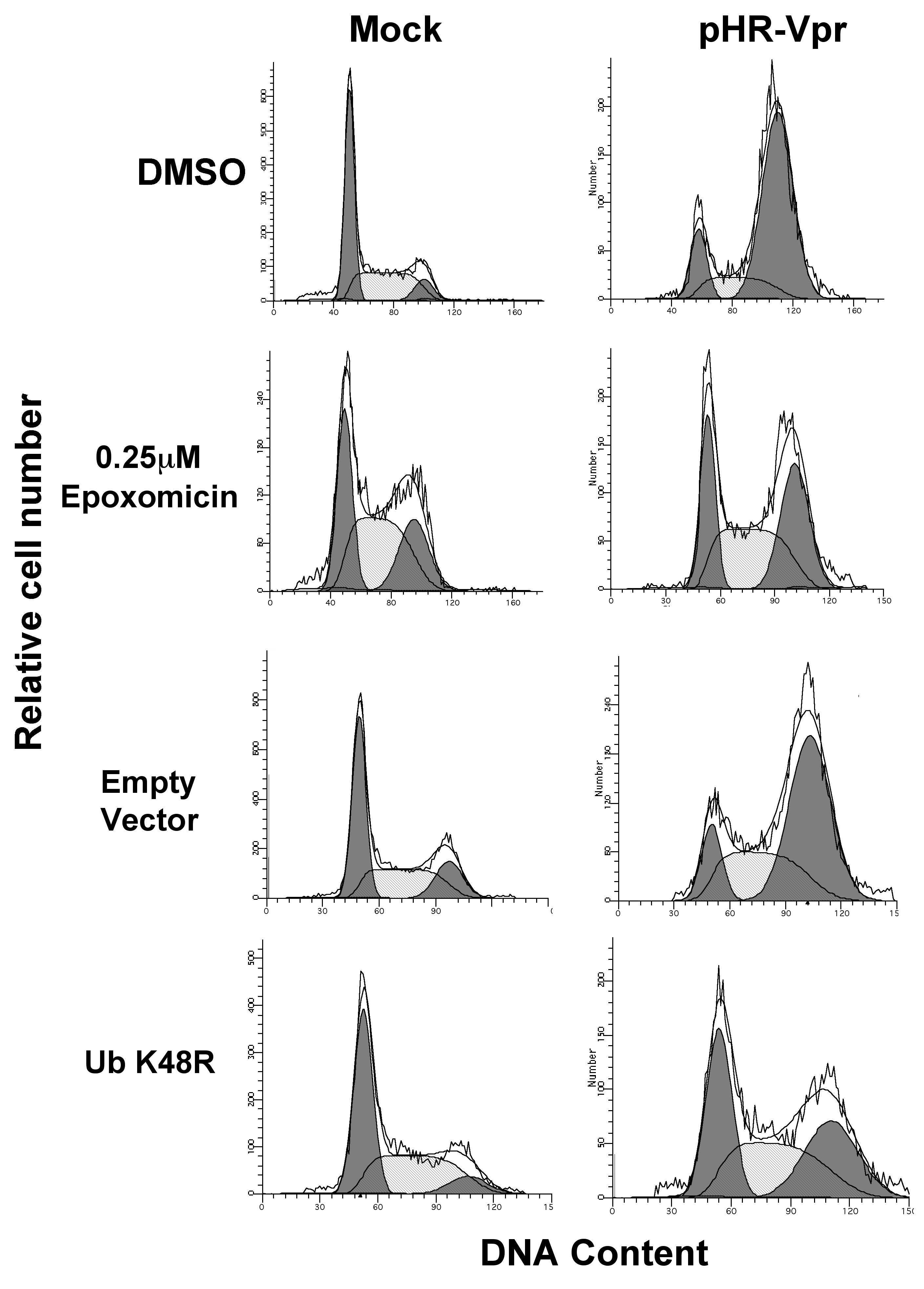

Supplement: Additional file 1 — Cell cycle profiles for experiments on the role of the ubiquitin proteasome system in Vpr-induced G2 arrest, corresponding to data shown in Figure 1. [file 1743-422X-4-57-S1.jpeg]

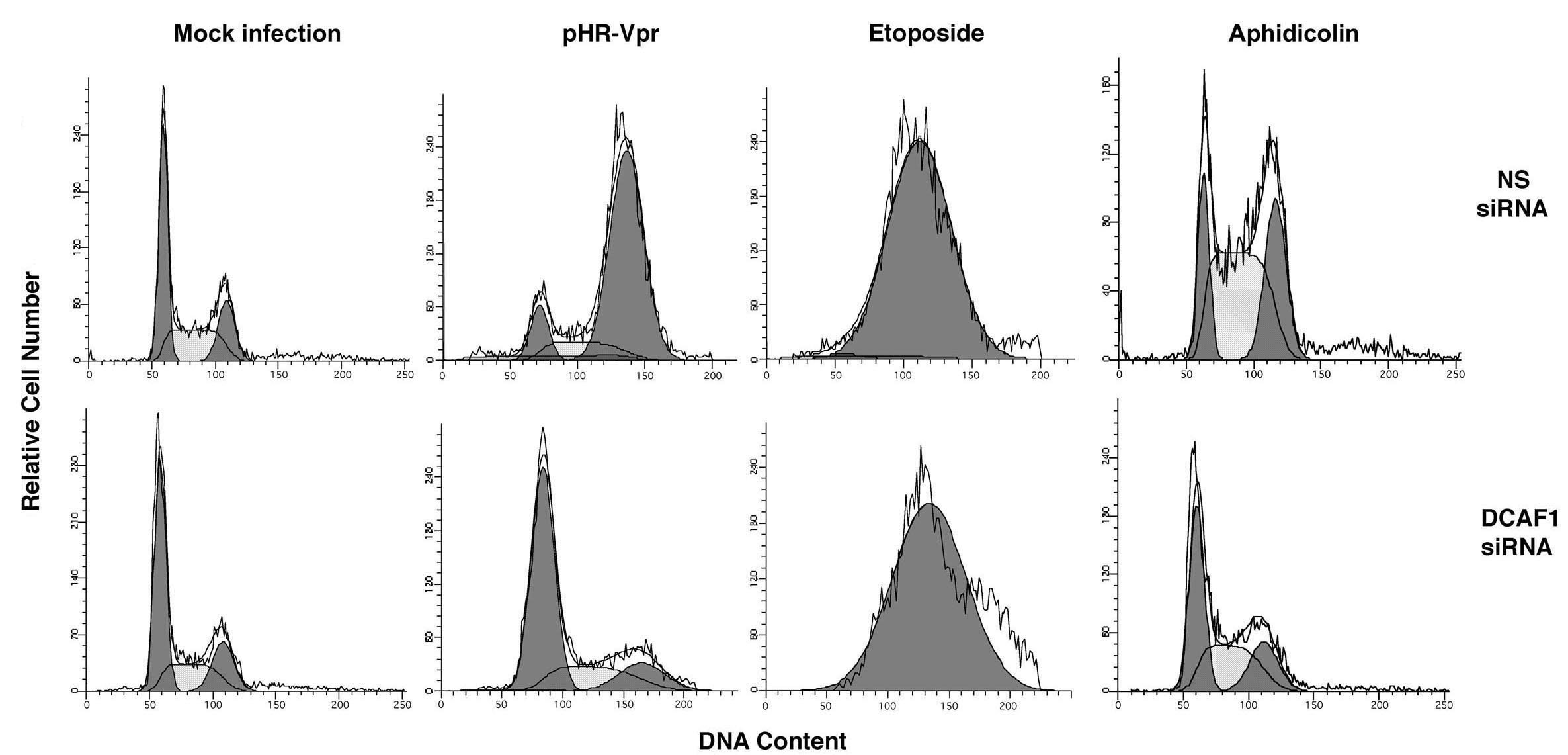

Supplement: Additional file 2 — Cell cycle profiles for experiments on the role of DCAF1 in Vpr-, etoposide- and aphidicolin-induced G2 arrest, corresponding to data shown in Figure 2B. [file 1743-422X-4-57-S2.jpeg]

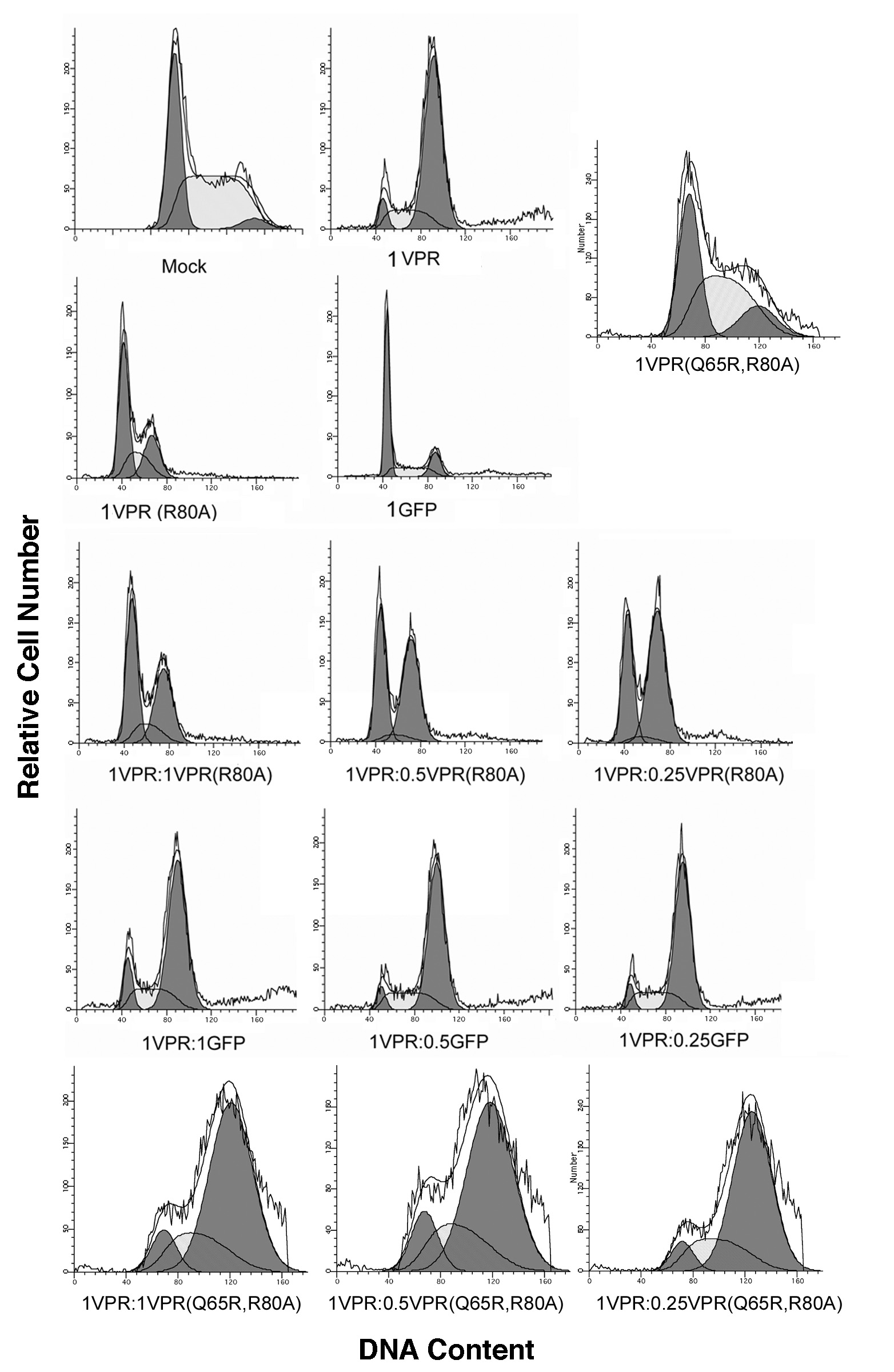

Supplement: Additional file 3 — Cell cycle profiles for experiments showing the dominan-negative activity of Vpr(R80A), corresponding to data shown in Figure 4. [file 1743-422X-4-57-S3.jpeg]
